# Pre-pubertal bipolar disorder: origins and current status of the controversy

**DOI:** 10.1186/s40345-020-00185-2

**Published:** 2020-04-20

**Authors:** A. Duffy, G. Carlson, B. Dubicka, M. H. J. Hillegers

**Affiliations:** 1 Queen’s University, Kingston, Canada; 2 Department of Psychiatry, University Oxford, Oxford, UK; 3 Renaissance School of Medicine, Stonybrook University, Stony Brook, NY USA; 4 Faculty of Biology, Medicine and Health, University of Manchester, Manchester, UK; 5 Department of Child and Adolescent Psychiatry and Psychology, Erasmus Medical Center-Sophia Children’s Hospital, Rotterdam, The Netherlands

**Keywords:** Bipolar disorder, Pre-pubertal bipolar disorder, High-risk, Epidemiology, Cross-national, Debate, Controversy, Diagnosis

## Abstract

**Background:**

Evidence from epidemiological, clinical and high-risk studies has established that the peak period of risk for onset of bipolar disorder spans late adolescence and early adulthood. However, the proposal of the existence of a pre-pubertal form of bipolar disorder manifesting in early childhood created substantial debate. In this narrative review, the literature and contributing factors pertaining to the controversy surrounding the proposed pre-pubertal bipolar disorder subtype are discussed. The resolution of the debate and lessons learned are highlighted.

**Main body:**

In the mid 1990s US researchers proposed that chronic irritability and explosive temper in pre-pubertal children with pre-existing ADHD and/or other learning and developmental disorders might represent a variant of mania. A number of factors contributed to this proposal including severely ill children with no diagnostic home given changes in the ADHD DSM diagnostic criteria and over-reliance on symptoms and structured interviews rather than on a clinical assessment incorporating developmental history, social context and clinical course. Prospective studies of children at high familial risk did not support the proposed pre-pubertal bipolar phenotype; but rather provided convergent evidence that bipolar disorder onset in adolescence and early adulthood not uncommonly preceded by sleep and internalizing symptoms and most often debuting as depression in adolescence (after puberty). Epidemiological studies of population and hospital discharge data provided evidence that the pre-pubertal bipolar phenotype was largely a US driven phenomenon.

**Conclusions:**

Psychiatric diagnosis is particularly challenging given the current lack of objective biomarkers. However, validity and utility of clinical diagnoses can be strengthened if all available predictive information is used to formulate a diagnosis. As in other areas of medicine, critical information required to make a valid diagnosis includes developmental history, clinical course, family history and treatment response—weighed against the known trajectories of classical disorders. Moreover, given that psychiatric disorders are in evolution over childhood and adolescence and symptoms, in of themselves, are often non-specific, a thorough clinical assessment incorporating collateral history and psychosocial context is paramount. Such an approach might have avoided or at least brought a more timely resolution to the debate on pre-pubertal mania.

## Background

As international clinician researchers who share a focus on bipolar and related mood disorders in children and adolescents, we have come together to update the status of the controversy surrounding the diagnosis of bipolar disorder in pre-adolescent children. The aim of this paper is to succinctly summarize what factors led to the proposal of a pre-pubertal or very early onset subtype of bipolar disorder and discuss key evidence that has helped to inform the debate. In the concluding remarks, we provide our collective thoughts more broadly on the lessons learned and future priorities for understanding psychopathology and the developmental trajectories of mental illness in young people.

## Origins of the controversy

Authors are required to disclose conflicts of interest. However, given the degree of discordance and debate, it would be helpful to also have a disclosure whereby authors or reviewers state what they fundamentally believe bipolar disorder is—especially in young children. In the mid to late 1990s a pre-pubertal bipolar subtype was proposed characterized by chronic irritability and explosive temper (taken as a manic equivalent) in the context of neurodevelopmental and externalizing behavior problems. “*Mania”* in these chronically ill children began between 4 and 7 years of age with estimated “*episode”* duration of 3+ years on average at the time of diagnosis; moderate to severe interference in functioning (CGAS under 50) and a high rate of comorbid externalizing disorders and learning problems (Wozniak et al. 1995; Geller et al. 2002; Tillman et al. 2003) were also characteristic. The proposal raised the question as to whether the definition of bipolar disorder should be restricted to the presence of discrete mood episodes that represent a clear departure from a person’s prior functional and clinical state or be broadened to include extremes of mood regardless of the duration or change from baseline. There was the additional question around whether a pediatric clinical subtype of an illness should have at least some continuity with the adult form of the illness. Further complicating the effort to validate the proposed pre-pubertal bipolar subtype was the fact that the criteria and structured interviews that were supposed to yield consistent samples didn’t (Duffy et al. 2018a). Thus, people reading articles from various research groups might assume that the samples being described reflected the reader’s view of what bipolar disorder is—which is not necessarily the case.

A case in point is a recently published genetic study on the association of genetic and environmental risks for attention deficit hyperactivity disorder (ADHD) with hypomanic symptoms in youth (Hosang et al. 2019). The findings hinge on two questionnaires that were given to assess hypomania—the Child Mania Rating Scale-Short Form (Henry et al. 2008) and the Mood Disorders Questionnaire (Wagner et al. 2006). These instruments have been said to distinguish bipolar disorder from ADHD. However, a closer look at these studies reveals that the children identified as having bipolar disorder might be among those children now re-conceptualized as having disruptive mood dysregulation disorder (DMDD) or affect lability related to some other problem or condition. For instance, there is a developing body of literature that recognizes the fact that ADHD in childhood is indeed sometimes complicated by significant affective lability (Shaw et al. 2014; Faraone et al. 2019) and that this may be mistaken for bipolar disorder (Pataki and Carlson 2013). Questionnaires cannot magically solve this problem. A different interpretation of the purported genetic association between ADHD and bipolar disorder would argue that there is a genetic association because in these children they are two forms of the same condition. If one is to make any sense out of a particular article, then, it is important to know who, exactly, comprises the sample.

Carlson and Klein (2014) have examined reasons why there have been such divergent views regarding the pre-pubertal or very early onset bipolar disorder diagnosis:

i. *ADHD is confused with mania*, that is many children with ADHD have a problem with low frustration tolerance, currently labelled *irritability* or *mood dysregulation,* was ignored. If mood lability is considered to be unique to bipolar disorder, then a group of children that some might diagnose with ADHD will be given a bipolar diagnosis by others. This goes for their families too, since such traits may well be familial (Saudino 2005).
ii. A *poor definition of distinctive episodes for mania* increased the emphasis on symptoms to make the diagnosis. Episodes were defined by symptoms of “*at least a week*” but that definition did not require an offset of symptoms thus leading to “*manic*” episodes in children that were typically years in length (Geller et al. 2004).
iii. *Irritability, a cardinal symptom of the proposed pre*-*pubertal mania criteria, was never defined.* It was initially present as part of depression in DSM III (“*dysphoric mood is characterized by symptoms such as the following: depressed, sad, blue, hopeless, low, down in the dumps, irritable*” p. 273) but was eliminated from adult depression criteria in DSM III-R. It was kept as a symptom of child depression—but not without some disagreement (Stringaris et al. 2013). DSM III-R recognized that adjustment disorder and oppositional defiant disorder could present with irritability (p. 536)—again not defined. The term *irritability* is never used in conjunction with ADHD, but starting in DSM-III, under associated features, references to “*increased mood lability, low frustration tolerance, temper outbursts, low self*-*esteem*” can be found. Therefore, in children, irritable mood with symptoms of ADHD could easily be construed as a “*mixed episode*” (Wagner et al. 2006).
iv. The *absence of developmentally informed criteria* in DSM III-IV added to the confusion. For instance, the symptoms of ADHD are defined: *six (or more) … symptoms of hyperactivity impulsivity [that are] inconsistent with developmental level*. Unfortunately, we don’t have developmentally informed information about manic symptoms e.g. we don’t know what constitutes” *developmentally appropriate euphoria*”. Little children can be silly in ways that would look inappropriate in an adult. Little children can say things that in an adult would sound grandiose (Carlson and Meyer 2006).
v. There has been *no consistent approach to manage information from multiple informants*. This is especially problematic in child psychiatry because diagnoses differ depending on whether everyone’s positive symptoms are counted even if inconsistent versus counting only those where there is agreement between informants. Rather than trying to understand what the discrepancy tells us, the approach has been to count one or the other informant or combine everyone’s positive symptoms. Such diagnostic approaches can be highly susceptible to confirmatory bias. For example, disparate teacher information in the Longitudinal Assessment of Manic Symptoms (LAMS) study (Horwitz et al. 2010; Findling et al. 2010) was ignored because it didn’t agree with interview diagnoses or parent ratings—instead it was parents’ information that counted toward a diagnosis. If teachers observe no evidence of irritability, hyperactivity, rapid speech etc. where parents rate the child as severely ill, the discrepancy needs to be understood. Mania does not just occur at home.
vi. *Equating all structured or semi*-*structured interviews* even though they actually collect different information (Galanter et al. 2012).
vii. *Double counting symptoms* so that someone meets criteria for two disorders with one set of symptoms or thinking one can avoid the problem simply by not counting the overlapping symptoms (Wagner et al. 2006).

Even with the unintended downstream effects of DSM criteria and shifts in diagnostic approaches, had the concept of bipolar disorder in very young children not met a clinical need it might not have become an accepted or popular diagnosis. There was and continues to be a need to reach an understanding as to how to conceptualize and treat children with explosive, aggressive behavior who do not fit typical cases of ADHD, conduct disorder, pervasive developmental disorder or other conditions in which aggression plays a role (Carlson and Klein 2018). Further, a recent systematic review suggests that prepubertal mania preferentially responds to antipsychotics (often with concurrent stimulant medication) rather than to lithium or anticonvulsants (Duffy et al. 2017a). Recently, explosive behavior has been subsumed under the umbrella of chronic irritability with Leibenluft and the NIMH developing an extensive study of irritability in children. Originally called “*severe mood dysregulation*” the condition was defined by chronic irritability with rages and hyperarousal and impairing symptoms of either in at least two settings (Leibenluft et al. 2003).

Chronic irritability is no longer felt to be a harbinger or alternative form of bipolar disorder. One definition of irritability is “*proneness to anger compared with peers at the same developmental level*” and it cuts across diagnoses (Stringaris et al. 2018). However, this has its own short-comings as irritability severity is determined in most studies only by the frequency of temper loss. The frequency and intensity, duration and context of the resulting explosive behavior (Carlson and Klein 2018) has not been adequately operationalized. To accurately reflect the behaviors that cause impairment, it will be necessary for measures to address the resulting verbal and physical aggression—what the child does when irritated. Otherwise, there will be no way to meaningfully distinguish unpleasant, grouchy children from those with significant explosive temper and aggression—those same children previously at the centre of the pre-pubertal bipolar diagnostic controversy.

## Longitudinal high-risk studies

Bipolar disorder is highly heritable (McGuffin et al. 2003) and relatives of bipolar patients are at an increased risk of developing bipolar and related mood disorders. While specific estimates vary between families and studies, convergent evidence supports that first degree relatives of an adult with bipolar disorder have an 8–10-fold risk of developing bipolar disorder and a 2–3-fold risk of developing unipolar disorders compared to the general population or low-risk controls (Duffy et al. 2000). The risk is even higher for children with both parents affected (Gottesman et al. 2010). Therefore, children of bipolar parents are an important high-risk group that can inform the onset and early course of bipolar disorder. Moreover, children at confirmed familial risk would be the most likely to manifest early onset subtypes thus shedding light on the controversy surrounding the validity of the proposed pre-pubertal bipolar phenotype. The question therefore is *does the proposed pre*-*pubertal bipolar phenotype occur in children at confirmed familial high*-*risk*?

There have been several published studies, narrative reviews and meta-analyses informing the prevalence and age of onset of psychopathology in children of variably identified and diagnosed samples of bipolar parents published over the last few decades (Duffy et al. 2011, 2017b; DelBello and Geller 2001; Lau et al. 2018; Lapalme et al. 1997; Ellersgaard et al. 2018). Several of these studies have repeatedly assessed high-risk children through the peak period of risk from childhood into early adulthood (Mesman et al. 2013; Duffy et al. 2018b; Axelson et al. 2015; Preisig et al. 2016; Egeland et al. 2012). While methods of identifying and assessing parents and children vary (Duffy et al. 2011), all have reported an increased risk for mood disorders in general, with depressive disorders more prominent than bipolar disorder. For example, the Dutch and Swiss studies reported lifetime rates of mood disorder of 54% and 63%, and lifetime rates of bipolar spectrum disorder of 13% and 17% respectively, for offspring observed into early adulthood (mean age of 28 and 21 years). The BIOS study reported a lifetime rate of mood disorder of 48% and bipolar spectrum disorder of 23% by age 21. In the Dutch, Amish and Canadian studies, onset of clinically significant manic symptoms began in adolescence.

The Dutch and Canadian studies, reported that bipolar disorder debuted as a depressive disorder in the vast majority of cases (88% and 85%, respectively) with onset in mid-adolescence (15 and 17 years) and the first diagnosable activated episode (i.e. hypomanic or manic) following years later (5 years on average) (Mesman et al. 2013; Duffy et al. 2014, 2018b). Further, the Dutch study found that in over one-third of offspring who developed bipolar disorder the preceding depressive disorder was recurrent (not chronic) in nature (Mesman et al. 2013). Similarly, the Canadian study found that the first five mood episodes of diagnosed bipolar disorder in high-risk offspring were predominantly depressive (not manic) (Duffy et al. 2018b). The age of onset for bipolar disorder was 20.7 years (range 12.4–30) in the Canadian cohort and 19.4 years (range 13–25) in the Dutch cohort. Similarly, the Amish high-risk study reported a median age of onset of mania of 18 years (range 13–29 years) (Egeland et al. 2012). The BIOS study reported a younger mean age of onset of mania or hypomania at 13.4 ± 3.8 years (Axelson et al. 2015). While no high-risk children met diagnostic criteria for bipolar disorder prior to age 12 in the Amish, Dutch or Canadian studies (502 total subjects) (Mesman et al. 2013; Duffy et al. 2018b; Egeland et al. 2012), the BIOS study reported that 13 of the 15 children manifest the first manic episode prior to age 12 (Axelson et al. 2015). Factors that might explain this discrepancy include differences in the BIOS parent sample (i.e. high rates of comorbidity including substance abuse, lower familial SES, assortative mating and ascertainment bias) (Duffy et al. 2011), as well as cross-national differences in diagnostic conceptualization of psychopathology in young children discussed below (Mesman et al. 2016; Dubicka et al. 2008).

Longitudinal high-risk studies have not only been helpful in characterizing the onset and early course of bipolar disorder, but also in characterizing early childhood functioning and clinical antecedents. The Dutch study reported normal or good overall level of social functioning in high-risk offspring compared to the general population from ages 11–18 years (Reichart et al. 2007). The onset of mood disorders in this cohort was associated with lower scores in family and adaptive functioning compared to the general Dutch population, but with small effect sizes (1.8% and 1.3% respectively). The Canadian study reported normative or gifted childhood functioning (social and academic) in the offspring of lithium responders (proxy for classical manic depressive illness) compared to offspring of healthy controls, and somewhat lower early childhood functioning on average in the offspring of lithium-non-responders (proxy for heterogeneous bipolar subtype) (Duffy et al. 2002). At last assessment, the global assessment of functioning (GAF) scores were stable in well or remitted offspring of lithium responders (median GAF 85), but had declined from baseline in the well or remitted offspring of lithium non-responders (median GAF 80). Further, there were comparable rates of ADHD and disruptive behaviour disorders (DBD) to the general population and low-risk comparison groups in the Dutch, Amish and Canadian high-risk offspring studies. The BIOS study reported significantly higher rates of ADHD and DBD in high-risk offspring compared to controls; however this difference diminished when adjusting for confounds related to the general level of family psychopathology and dysfunction (Birmaher et al. 2009).

Based on longitudinal observations into adulthood, there have been recent efforts to model the developmental trajectory of emergent bipolar disorder in children at confirmed familial risk (Duffy et al. 2014, 2018b) and to advance individualized risk prediction (Hafeman et al. 2016). Based on the Canadian data, as a general model of the developmental trajectory of bipolar disorder supported a progressive sequence of psychopathology; shifting from sleep and anxiety symptoms and disorders in childhood to minor depressive and adjustment disorders (internalizing symptoms related to stressors) in early adolescence (around or after puberty) to major depressive disorder and hypomanic symptoms in mid-adolescence and to bipolar disorder in late adolescence and early adulthood (Duffy et al. 2014, 2018b). The transition from depression to bipolar disorder was more likely if depression was recurrent and/or included psychotic symptoms within the episode. Also, substance abuse onset earlier in high-risk offspring and in close proximity to the onset of depressive disorders (Duffy et al. 2012, 2014). Based on BIOS data, baseline (childhood) parent-reported anxiety and depressive symptoms and child-reported mood lability together with more proximal (to the first manic episode) mood lability and hypomanic symptoms predicted new-onset bipolar spectrum disorders. In the BIOS, Canadian and Swiss studies, earlier onset of parental BD was associated with a higher risk of developing bipolar disorder (Duffy et al. 2018b; Preisig et al. 2016; Hafeman et al. 2016).

**Fig. 1:**
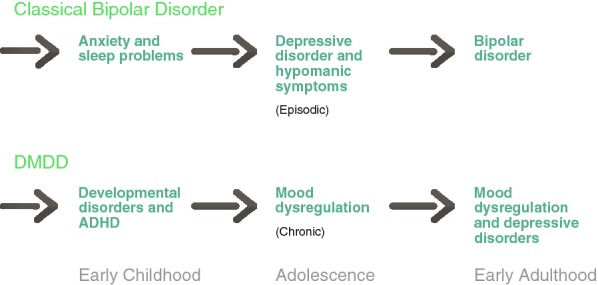
Developmental trajectory of bipolar disorder vs severe mood dysregulation or disruptive mood disregulation disorder

In summary, several independent longitudinal prospective studies of children of parents diagnosed with bipolar disorder according to DSM or ICD criteria, have provided important and mostly convergent insight into the onset and early course of bipolar disorder that do not align with the proposed pre-pubertal bipolar subtype hypothesis. Key findings in high-risk children include that (i) premorbid social and cognitive/academic functioning is comparable to the general population; (ii) childhood internalizing symptoms and disorders and sleep problems but not neurodevelopmental, cognitive or externalizing disorders predict for onset of mood disorder in adolescence and early adulthood; (iii); bipolar disorder typically debuts with depressive episodes with a recurrent illness course starting in adolescence; (iv) psychotic symptoms in depressive episodes predict for conversion to bipolar disorder; (v) clinically significant sub-threshold manic symptoms (often episodic) have a variable age of onset (typically not pre-adolescent), and predict for subsequent onset of bipolar disorder in emergent adulthood. Finally, and relevant to the bipolar controversy, the so-called pre-pubertal bipolar phenotype has not been observed in children of parents with a confirmed bipolar diagnosis in the vast majority of studies and is therefore consistent with a different illness trajectory (Fig. 1).

It is important to note that substantial differences in the quality of remission, nature and rates of comorbid disorders, as well as global functioning in subgroups of high-risk children have been reported. For example, in the Canadian study there were significant differences in the quality of early childhood functioning (academic and social), nature of non-mood comorbidity (i.e. sequential vs concurrent), quality of mood disorder remission, and spectrum of end stage disorders in the offspring of parents with a classical bipolar disorder responsive to long-term lithium compared to the offspring of parents with a less typical lithium-non-responsive psychotic subtype of bipolar disorder (Duffy et al. 2009, 2018b). Therefore, heterogeneity of our current diagnoses, including bipolar disorder, require more detailed attention if we hope to understand stable developmental trajectories, map reliable biomarkers and develop specific effective treatments. This notwithstanding, there was no evidence of a pre-pubertal form of bipolar disorder across these high-risk subtypes.

## Cross-national differences

The debate around the diagnosis of bipolar disorder in pre-pubertal children ignited in the 1990s with the publication of data from a US tertiary care centre suggesting that up to 40% of children with ADHD also exhibited “mania” (Wozniak et al. 1995; Biederman et al. 1999). These reports caused significant debate as findings were not replicated in several centres outside the US. For example, using a nationwide psychiatric case register in Denmark covering a background population of 5.1 million inhabitants, 39 children (23 boys, 16 girls) were identified has having the diagnosis of manic-depressive psychosis between 1970 and 1986 before the age of 15 (Thomsen et al. 1992). In addition, no cases of mania were found in a British clinic survey of 2500 children aged 10 and under (Harrington and Myatt 2003) and only one child met diagnostic criteria for both ICD–10 hypomania and DSM–IV bipolar disorder NOS in a UK sample of 200 young people with ADHD (6–18 years, mean age 11.15) (Hassan et al. 2011). A recent meta-analysis re-examined prevalence rates of paediatric bipolar disorder in epidemiological samples (Van Meter et al. 2019). The authors concluded that there was no difference in rates of paediatric bipolar disorder in the US compared to other countries based on data from structured interviews in epidemiological populations. However, the studies included mostly adolescent not pre-pubertal subjects and there was evidence of significant heterogeneity likely associated with bipolar subtype (I, I or II, spectrum), age, sample size, diagnostic criteria and structured interviews scoring externalizing behaviour as a bipolar equivalent (Parry et al. 2019a).

Therefore, questions arose regarding cross-national or other factors influencing clinician diagnosis of pre-pubertal bipolar disorder, previously recognised in conditions such as schizophrenia (Gupta 1992) and ADHD (Prendergast et al. 1988). A US–UK cross-national study investigated potential diagnostic biases as a source for the discrepancy in prevalence rates (Dubicka et al. 2008). Five vignettes were presented to UK and US clinicians, four representing complex scenarios where the diagnosis of mania was thought to be controversial and one a ‘classical’ case of mania in an adolescent where it was expected there would be good agreement. As predicted, overall US clinicians were significantly more likely to diagnose mania than UK clinicians in the younger complex cases, but there was good agreement on classical mania in the adolescent case. UK clinicians were significantly more likely to diagnose pervasive developmental and adjustment disorders, while US clinicians were more likely to diagnose mania and additional comorbid disorders.

In a recent Dutch-US cross-national comparison study of psychopathology in offspring (aged 6–21 years) of parents with bipolar disorder, the inter-site reliability was measured using US audiotapes of a semi-structured psychiatric interview. Specifically, 5 selected audiotapes from the BIOS study assessments were blindly rated by 4 interviewers from Pittsburgh and 4 interviewers from the Netherlands (Mesman et al. 2016). This study indicated good agreement in assessing the narrow definition of bipolar disorder (BD I and II), but cross-national differences in assessing co-morbid externalizing disorders. Given that the BIOS sample were younger this might have impacted the rate of co-morbidity and age of onset of bipolar disorder. However reducing this possibility, the Danish High Risk and Resilience Study—VIA 7, a nationwide population-based cohort study of 522 7-year-old (age range 6.9–8.4 years) high-risk children (Ellersgaard et al. 2018), reported a higher prevalence of anxiety disorders, stress and adjustment disorders compared with controls and no cases of prepubertal bipolar disorder. Another cross-national study of mania in adults reported that UK psychiatrists were less likely to endorse symptoms on the Young Mania Scale than were clinicians from the US or India (Mackin et al. 2006). These studies provided evidence that with the same information the interpretation and thus diagnosis varied across clinicians from different countries.

Using outpatient treatment data, Moreno and colleagues showed a substantial increase (40-fold) in the rate of paediatric bipolar disorder diagnosis in the US between 1994 and 2003 (Moreno et al. 2007)—a trend that has not been replicated in the same way in other countries. For example, James and colleagues reported a 72.1 times higher hospital discharge rate between the US and England for bipolar disorder in children and adolescents, reduced to a 12.5-fold difference after adjusting for length of stay (James et al. 2014). The largest disparity in discharge rates occurred in the pre-adolescent period and by age 5 years the rate of discharge in the US was greater than the maximum discharge rate in England, which occurred at age 19. The study of hospital discharge rates for bipolar disorder was expanded to include other countries compared to the US (Clacey et al. 2015). For those under 20, the discharge rates for bipolar disorder per 100,000 population were: US 95.6, Australia 11.7, New Zealand 6.3, Germany 1.5 and England 0.9. The most marked difference was in 5- to 9-year-olds with per 100,000 population rates: US 27, New Zealand 0.22, Australia 0.14, Germany 0.03 and England 0.00. Interestingly the trend in discharge rates in the US showed a sharp peak in 5–9 year olds, while in the other countries there was a gradual rise in discharge rates starting from early adolescence (Clacey et al. 2015).

Cross-national differences in clinical practice guidelines and diagnostic criteria reflect the pre-pubertal bipolar diagnostic controversy (Parry et al. 2019b). In a review of published articles, among 624 articles with US authorship, the majority (83%) were found to support the validity of a pre-pubertal form of mania. Of 163 articles by non-US authors, most (60%) supported the traditional view that mania and therefore bipolar disorder is rare before mid-adolescence. DSM is based on symptom counting, whereas ICD, more commonly used outside the US, allows for a pattern recognition approach to diagnosis and has generally avoided arbitrary cut-offs and precise requirements regarding symptom counts. The ICD approach is intended to reflect the way clinicians make diagnoses (Reed et al. 2019). In addition, ICD-11 requires more than one manic episode for a diagnosis of bipolar disorder, whereas the DSM-IV-TR and the DSM-5 require only one such episode. To avoid over-diagnosis in children and adolescents, ICD-11 states that the diagnosis of bipolar disorder requires the presence of mania with unequivocal euphoria (not just irritability) and an episodic course.

Similarly, the UK National Institute for Health and Care Excellence (NICE) (National Collaborating Centre for Mental Health 2014) clinical practice guideline recommends conservative use of the diagnosis of bipolar I disorder in children and adolescents and cautions against making bipolar II disorder diagnoses in this age group. It also states that irritability should not be used as a core diagnostic criterion. Whereas some US centres have maintained that pre-pubertal bipolar disorder is characterised by non-episodic, chronic, ultra-rapid cycling, mixed/irritable states, in the UK, and perhaps elsewhere, such cases are more likely to be conceptualised as oppositional defiant disorder (ODD), conduct disorder and/or ADHD with emotional dysregulation (Sobanski et al. 2010). The ICD-11 ODD category includes a “*with chronic irritability and anger*” qualifier to characterize presentations with prevailing, persistent irritable mood or anger. This presentation is recognized to significantly increase the risk for subsequent depression and anxiety. The ICD-11 conceptualization of this presentation as a form of ODD diverges from the DSM-5 approach of introducing a new disorder “*disruptive mood dysregulation disorder*” highlighting international differences in the approach to this constellation of symptoms.

Unique aspects of the US health system may have been important in the greater acceptance of pre-pubertal *mania*  (bipolar disorder) as a valid diagnosis. It has been argued that the US health system often drives clinicians to engage in “*diagnostic upcoding*” and managed care has been anecdotally reported as providing more funding for a diagnosis like bipolar disorder, than for difficulties such as parent–child relational problems (Parry et al. 2019b). It has been argued that the expansion of bipolar disorder has been created in order to market new drugs into the more profitable realm of everyday emotional problems, rather than limiting them to classical forms of bipolar disorder, and in so doing, medicalizing personal and social difficulties (Moncrieff 2014).

Finally, there are cross-national differences in the general acceptance of diagnoses in children and a proclivity to not diagnose children with a mental disorder is more active outside than within the US (National Collaborating Centre for Mental Health 2014). In some countries, there is a policy movement towards a focus on mental well-being (which remains a nebulous concept) and away from mental illness. For example, the Netherlands is currently in the middle of a substantial decentralization and transformation of the Dutch youth care system including youth mental health (Sobanski et al. 2010). This transition tasks Dutch municipalities with the coordination of most services in the social domain and promotes de-medicalization—a concept that arises from philosophical and ideological driven frameworks, rather than robust scientific justification. This would potentially remove consideration of mental disorders as discrete entities and substitute existential experiences as the target for mental health care. How this approach will further or hinder access to best evidence-based care for those young people who require it, or how this approach will better assist us with developing necessary and effective treatments remains to be proven.

## Conclusions

Several different forces have contributed to the proposal that chronic aggression and explosive temper in very young children with comorbid ADHD and associated problems in social and academic functioning represented a pre-pubertal form of bipolar disorder. Although based on findings from clinical, high-risk and epidemiological studies, the pendulum is now swinging away from that point of view, we still have no consensus regarding the specificity of irritability or explosive behavior across different conditions, which was at the core of the pre-pubertal mania controversy. Disruptive mood dysregulation disorder, proposed as an alternative diagnosis for these children, is problematic in that explosive temper is defined by frequency rather than severity of outbursts and it is difficult to define “*mood between outbursts*” (Galanter et al. 2012). Moreover, it is not at all clear whether adding a comorbid diagnosis is more helpful than considering explosive temper and mood lability as subtype of ADHD denoted by a diagnostic specifier—coming full circle as it were and supported by convergent evidence that childhood ADHD is associated with psychotic and neurodevelopmental disorders (Hamshere et al. 2013a; b; MacCabe and Murray 2004; Murray et al. 2004). Nevertheless, this journey has been helpful in underscoring the importance of a comprehensive clinical assessment, which as in other areas of medicine should include collecting collateral history from multiple sources, distinguishing illness episodes from baseline functioning, and assessing symptoms in the context of the developmental history, family history, clinical course, treatment response and psychosocial context (Duffy et al. 2018a).

Longitudinal prospective studies of children at confirmed familial high-risk of developing bipolar disorder have not supported the validity of the pre-pubertal bipolar phenotype proposed by Geller et al. (2004), Wozniak et al. (1995) and others. This is important because bipolar disorder is highly heritable (McGuffin et al. 2003; Bienvenu et al. 2011) and thus children of affected parents would be the most likely group expected to manifest an early-onset prepubertal form of bipolar disorder—if it existed. This assumption is underscored by the fact that the Amish and Canadian high-risk families were selected initially for genetic studies owing to the high penetrance of bipolar illness across multiple generations.

The validity of the proposed pre-pubertal form of bipolar disorder has been a focus of significant debate and controversy stretching over two decades. Without reliable and objective biomarkers to validate clinical diagnoses differences of conceptualization and opinion are more likely and difficult to resolve. The corollary of the pre-pubertal mania controversy is the recognition that, while there are many instances of clinical uncertainty especially on the basis of a cross-sectional structured (symptom focused) assessments  of children with emerging clinical pictures, to improve our diagnoses we should rely upon what we know about prototypical developmental trajectories of severe mental illnesses. Combining developmental course, with the context of symptoms and family history of mental illness will help our assessment as to whether something is (i) diagnosable as a separate comorbid illness, (ii) part of a developing or evolving illness trajectory or (iii) a normative developmental variant or reaction to a specific psychosocial stressor or context and as such not diagnosable. Further, any proposed early-onset subtype should have proven association and continuity with the prototypical adult form—otherwise there is no basis for a relationship.

Diagnosis is a cornerstone of medical practice. Yet, there is growing recognition that the current practice of basing diagnoses on symptom checklists is highly problematic (Duffy et al. 2018a). As stated by Kendler “*since DSM III, our field has moved toward a reification of the DSM that implicitly assumes that psychiatric disorders are just the DSM criteria. That is, we have taken the index of something for the thing itself*” (Kendler 2016). Such an approach is insufficient for accurately defining an illness with precision, differentiating it from other illnesses and mapping the clinical illness to underlying pathophysiology in order to develop specific targeted treatments and refine prognostic predictions. Ultimately this debate profoundly matters because diagnoses have significant meaning and consequences for the individual and their family and for scientific advancement.

## Data Availability

All studies reviewed are published and available.
